# A Novel Blood-Brain Barrier Co-Culture System for Drug Targeting of Alzheimer’s Disease: Establishment by Using Acitretin as a Model Drug

**DOI:** 10.1371/journal.pone.0091003

**Published:** 2014-03-07

**Authors:** Christian Freese, Sven Reinhardt, Gudrun Hefner, Ronald E. Unger, C. James Kirkpatrick, Kristina Endres

**Affiliations:** 1 REPAIR-lab, Institute of Pathology, University Medical Center of the Johannes Gutenberg University Mainz and European Institute of Excellence on Tissue Engineering and Regenerative Medicine, Mainz, Germany; 2 Department of Psychiatry and Psychotherapy, Medical Center of the Johannes Gutenberg University Mainz, Mainz, Germany; National Center for Geriatrics and Gerontology, Japan

## Abstract

In the pathogenesis of Alzheimer’s disease (AD) the homeostasis of amyloid precursor protein (APP) processing in the brain is impaired. The expression of the competing proteases ADAM10 (a disintegrin and metalloproteinase 10) and BACE-1 (beta site APP cleaving enzyme 1) is shifted in favor of the A-beta generating enzyme BACE-1. Acitretin–a synthetic retinoid–e.g., has been shown to increase ADAM10 gene expression, resulting in a decreased level of A-beta peptides within the brain of AD model mice and thus is of possible value for AD therapy. A striking challenge in evaluating novel therapeutically applicable drugs is the analysis of their potential to overcome the blood-brain barrier (BBB) for central nervous system targeting. In this study, we established a novel cell-based bio-assay model to test ADAM10-inducing drugs for their ability to cross the BBB. We therefore used primary porcine brain endothelial cells (PBECs) and human neuroblastoma cells (SH-SY5Y) transfected with an ADAM10-promoter luciferase reporter vector in an indirect co-culture system. Acitretin served as a model substance that crosses the BBB and induces ADAM10 expression. We ensured that ADAM10-dependent constitutive APP metabolism in the neuronal cells was unaffected under co-cultivation conditions. Barrier properties established by PBECs were augmented by co-cultivation with SH-SY5Y cells and they remained stable during the treatment with acitretin as demonstrated by electrical resistance measurement and permeability-coefficient determination. As a consequence of transcellular acitretin transport measured by HPLC, the activity of the ADAM10-promoter reporter gene was significantly increased in co-cultured neuronal cells as compared to vehicle-treated controls. In the present study, we provide a new bio-assay system relevant for the study of drug targeting of AD. This bio-assay can easily be adapted to analyze other Alzheimer- or CNS disease-relevant targets in neuronal cells, as their therapeutical potential also depends on the ability to penetrate the BBB.

## Introduction

Alzheimer’s disease (AD) is a progressive degenerative disorder of the brain. While maximally 5% of all cases of this type of dementia are based on gene mutations [1], the cause of the sporadically occurring cases is still enigmatic. Literature suggests an involvement of processes such as impairment of the blood-brain barrier (BBB), mitochondrial dysfunction and tau-mediated destabilization of microtubules [2], [3], [4]. Nevertheless, deregulation of the proteolytic processing of a type I transmembrane protein – the amyloid precursor protein (APP) – has been accepted as closely correlated to AD pathology. Therefore, interference with one of the proteinases that cleave APP offers a target for therapeutic strategies (e.g. reviewed in [5], [6], [7]. In the non-amyloidogenic pathway the alpha-secretase ADAM10 prevents formation of toxic A-beta peptides from APP and alternatively gives rise to a neuroprotective and neurotrophic soluble fragment (APPs-alpha) [8], [9], [10]. We were able to demonstrate that overexpression of ADAM10 in transgenic mice [11] and acitretin-induced upregulation of ADAM10 gene expression in an AD mouse model [12] leads to a significant reduction of A-beta peptides. Acitretin is an already FDA-approved drug for treatment of psoriasis and has been shown to penetrate into the brain of rats [13]. It does not show P-glycoprotein (P-gp) substrate properties as well as favorable kinetics [14] and therefore was directly applicable for entering a clinical study in humans (NCT01078168). To evaluate novel, potent alpha-secretase enhancers it has to be guaranteed that the drug candidates can cross the BBB and that they can act on central nervous APP processing. Overall, literature is heterogeneous regarding a disturbed BBB permeability in AD pathology [15]. On one hand, microvascular injury has been correlated with progress of disease pathology (Braak stages) and ApoE genotype [16]. On the other hand, albumin ratio (QAlb), which is an indicator of BBB function, showed no systematic differences compared within different ApoE genotype carriers [17]. However, an early treatment to prevent pathogenesis of AD is an urgent requirement for a drug with therapeutic value. A recent study of Vos and colleagues demonstrated that subjects with preclinical AD had a higher risk for development of AD [18]. Therapeutic intervention at such preclinical stages has to face the challenge of an unimpaired BBB, which basically is a tight barrier and transport of nutrients as well as drugs is highly regulated or even impaired due to cell-cell junctions and efflux transporters such as P-gp [19], [20], [21]. Primary *in vivo* screening processes for evaluating BBB permeability of drugs are time consuming and expensive. Thus, the establishment of *in vitro* models is under intensive investigation. A few well-characterized *in vitro* blood-brain barrier models have been described within the last years which were developed using primary endothelial cells from the brain of different species [22], [23], [24], [25]. Isolated from rat, bos taurus or pig, and co-cultured with astrocytes, pericytes or even both cell types, brain microvascular endothelial cells were shown to form a tight barrier, generally demonstrated by high transendothelial electrical resistance (TEER) and low permeability coefficients [26], [27], [28], [29]. In addition to primary isolated endothelial cells, immortalized cell lines such as HBMEC or hCMEC/D3 which express brain endothelial markers but exhibit lower TEER have also been used for establishment of such models [30], [31], [32], [33]. In general, the advantages of *in vitro* models are the possibility for high throughput screening, their reproducibility and more importantly, the reduction of animal experiments.

The aim of this study was the development of a post-screening bio-assay model for analyzing the transport properties of anti-AD drug candidates. It combines an *in vitro* BBB model system and a luciferase-based reporter assay to detect drug transported across the BBB model. For this purpose, we co-cultured porcine brain endothelial cells (PBECs) on filter membranes and human neuroblastoma cells (SH-SY5Y) transfected with an ADAM10-promoter-driven reporter gene, seeded on the bottom of the basal well. To make our bio-assay system easily available to other laboratories we also investigated if the cell line hCMEC/D3 can be used as the barrier building unit in this model. To validate the functionality of our bio-assay, the potent alpha-secretase enhancer acitretin was used as a model drug because it was shown to cross the BBB in rats and mice [13], [14] and to induce ADAM10 expression *in vivo* [12]. The benefit of such a co-culture system developed in this study is on the one hand the well-characterized BBB model and on the other hand the sensitive reporter gene-based detection of therapeutically active drug transported across the barrier.

## Materials and Methods

### Materials

Acitretin was purchased from LGC Promochem (Germany), DMSO and butylhydroxytoluol (BHT) from AppliChem (Germany).

### Isolation of Porcine Brain Endothelial Cells and Cell Culture

Brain microvascular endothelial cells (PBECs) were isolated from fresh porcine using a modified protocol which was described previously for the isolation of human brain microvascular endothelial cells [34]. Brains were provided by Mr. Wohn (local butcher, Mainz, Germany) and used with his permission. The meninges were carefully removed. The grey matter tissue was minced into small pieces and digested with 0.1% collagenase type IV (Worthington, NJ, USA) and 200 µl DNase I (100 µg/mL; Sigma-Aldrich, USA) for 30 minutes at 37°C. The tissue solution was diluted with PBS containing 20% Percoll Plus (GE Healthcare, Sweden) and centrifuged at 2600 rpm at 4°C for 1 hour. The capillary fragments were washed with PBS and digested with 1 mg/mL collagenase/dispase (Roche, Germany) and 150 µl DNase I at 37°C for 10 minutes. After additional washing with PBS, the cell pellet was resuspended in PBS and loaded on a prepared Percoll gradient [34]. Finally, cells were resuspended in ECBM, supplement mix (both PromoCell, Germany), penicillin/streptomycin (10,000 U/mL/10,000 µg/mL; Gibco, Germany), 3 µg/mL puromycin (Calbiochem, Germany), and seeded on fibronectin-coated HTS Transwell-24 polyester filter membranes (0.4 µm pore size, 6.5 mm in diameter; Corning Costar, USA). Cells were sustained in medium containing 3 µg/ml puromycin for 3 days. Afterwards, medium without puromycin was used and the transendothelial electrical resistance (TEER) was measured, beginning from day 6 of preparation. Cell experiments were started at day 8 with cells which exhibited a resistance of at least 170 Ω×cm^2^.

SH-SY5Y cells (neuroblastoma cell line; ATCC (Manassas, USA); # CRL-2266) were cultivated in DMEM/HamF12 medium (Gibco, Germany) supplemented with 10% FCS and 1% glutamine (both PAA, Germany). The human cerebral microvascular endothelial cell line hCMEC/D3 was kindly provided by Pierre-Olivier Couraud (Department of Cell Biology, Institut Cochin, Paris, France) [33]. hCMEC/D3 were cultivated on fibronectin-coated tissue culture flasks in ECBM, supplement mix and penicillin/streptomycin. The immortalized cell lines were passaged twice a week and maintained under standard conditions (5% CO_2_, 95% humidity, 37°C).

### Setup of the Co-culture Model System

hCMEC/D3 (10,000 cells/filter) were seeded on fibronectin-coated Transwell filters (Corning Costar, USA; see above) and cultured using ECBM, 15% FCS, 2.5 ng/mL basal fibroblast growth factor, 10 µg/mL sodium heparin (both Sigma-Aldrich, USA) and penicillin/streptomycin. At day 5 after seeding of hCMEC/D3, 250,000 SH-SY5Y cells were placed on the bottom of a 24-well plate compatible with the Corning Transwell filter plate using Opti-MEM (Invitrogen, Germany). For transport experiments with the test substance acitretin, SH-SY5Y cells were transfected with a human ADAM10-promoter luciferase reporter construct [12] as described in the respective section. After 5 hours the medium of SH-SY5Y cells was changed to DMEM/HamF12 medium supplemented with 10% FCS and 1% glutamine. Filters with hCMEC/D3 were put on top of the 24-well plate and cells were grown in co-culture for 2 days.

For the co-culture with PBEC, SH-SY5Y cells were seeded at day 8 after isolation of the primary cells by the same procedure as described for hCMEC/D3.

### Treatment of Cells with Acitretin

The co-culture of PBEC and ADAM10-promoter reporter transfected SH-SY5Y cells was set up as described above. For the application of acitretin, 50 µl of the supernatant of the upper compartment were replaced by acitretin diluted in ECBM, 15% FCS, 2.5 ng/ml basal fibroblast growth factor, 10 µg/ml sodium heparin (both Sigma-Aldrich, USA) and penicillin/streptomycin. The final concentration of acitretin in the upper compartment was 12 µM (in whole medium volume: 2 µM) and the cells of the co-culture model were incubated for 48 hrs. Mono-cultures of PBEC were compared to the co-culture. Empty transwell filters coated with fibronectin were used as controls in mono-cultures of SH-SY5Y cells. Dimethylsulfoxide (DMSO) served as the solvent control. TEER was measured before and after the treatment to ensure the tightness of the endothelial cell barrier. For experiments examining the cellular uptake of acitretin in brain endothelial cells, a final concentration of 2 µM acitretin was applied to the cells seeded in 24 well plates which were subsequently incubated for 48 hours.

### Detection of Acitretin by HPLC

To determine acitretin concentrations in the upper and lower compartment of the Transwell system, cell supernatant was collected at the end of the incubation period. The retinoid was stabilized by addition of BHT (50 µg/ml) and quantified by HPLC as described previously [14].

### Transfection and Reporter Gene Assays

Analysis of cellular uptake of acitretin into the brain endothelial cells was performed by transfection of a retinoid-response reporter with Lipofectamine LTX (Invitrogen, Germany). Briefly, 45,000 cells were seeded on 96 well plates in OptiMEM (Invitrogen, Germany) and transfected with 100 ng DR5 element reporter vector [12] using 0.5 µl transfection reagent per well as recommended by the manufacturer.

For co-culture experiments 250,000 SH-SY5Y cells were transiently transfected with 800 ng ADAM10-promoter reporter plasmid [12] in 24 well format using Lipofectamine 2000 (Invitrogen, Germany). The transfection procedure was performed as specified by the manufacturer. Cells were lysed after the incubation period with the appropriate lysis buffer (Promega, Germany) and light emission measured upon addition of luciferase substrate (Promega, Germany) using a Fluostar Omega (BMG Labtech). Protein content of the cell lysates from the reporter gene assays was determined with Nanoquant (Roth, Germany) and used for normalization of luciferase activity yielding the parameter, RLU (relative light unit).

### Fluorescence Imaging of Tight Junction Proteins

After incubation, endothelial cells attached to the filter membranes were washed with PBS (Gibco, Germany) and fixed with a mixture of methanol/ethanol (2∶1) at room temperature for 20 min. Cells were washed and stained with antibodies recognizing different tight junction proteins (zonula occludens protein-1, occludin (both Zymed Laboratories, CA, USA), claudin-5 (Abcam, UK)) and the corresponding secondary antibodies (Alexa fluor 546; Molecular Probes, CA, USA). All antibodies were diluted in 1% bovine serum albumin (Roth, Germany) in PBS. Nuclei were stained with Hoechst 33342 dye (Sigma-Aldrich, USA). The filter membranes were embedded with GelMount (Biomeda, Natutec, Germany) and analyzed via fluorescence microscopy (Olympus IX71 with Delta Vision system, Applied Precision, USA).

### Electron Microscopy

For electron microscopy analysis PBECs cultured on filter membranes were fixed with cacodylate-buffered glutaraldehyde (pH 7.2, Serva, Germany) for 20 minutes at room temperature. This was followed by a fixation step in 1% (w/v) osmium tetroxide for 2 hours and dehydration in ethanol. Cells were transferred through propylene oxide, embedded in agar-100 resin (PLANO, Germany) and polymerized at 60°C for 24 hours. Ultrathin sections were cut with an ultramicrotome (Leica Microsystems, Germany), placed onto copper grids and analyzed with a transmission electron microscope (Jem-1400, JOEL, Japan).

Samples for scanning electron microscopy were dried after the fixation step with osmium tetroxide (Sigma-Aldrich, St. Louis, MO; USA). They were transferred to a carbon-coated metal plate, sputtered with gold and analysed with a scanning electron microscope (Zeiss, Modell DSM 962).

### Histological Examination of Endothelial Cells Grown on Filter Membranes

PBECs cultured on filter membranes were fixed with 3.7% paraformaldehyde at room temperature for 15 minutes. Afterwards, cells were embedded in paraffin, thin sections were cut and stained with hematoxylin-eosin (Merck, Germany). Light microscopy was performed using a Biorevo BZ-9000 microscope (Keyence, Germany).

### Western Blotting

Cells were lysed in LDS sample buffer (Invitrogen, Germany) including 100 mM dithiothreitol (Roth, Germany) and protease inhibitor mix (Roche, Germany). 20 µg proteins of whole cell lysate were separated on 10% SDS-acrylamide gels and transferred to a nitrocellulose membrane. Blots were either blocked with 5% BSA or milk powder and incubated with primary antibodies diluted in respective blocking buffer as follows: anti-APP (previously described: [35]), anti-ADAM10 (Merck, Germany), anti-GSK3-beta (Bioss, Germany), anti-Pgp (Santa Cruz, Germany) anti-Actin (Sigma, Germany), anti-P-ERK and anti-GAPDH (both: Cell Signaling, USA). Detection of APPs-alpha was performed as a dot blot with direct application of cell culture supernatant to the nitrocellulose membrane and 6E10 (Covance, Germany) as primary antibody. Blots were incubated with respective HRP-labeled secondary anti-mouse or anti-rabbit antibodies (Thermo Scientific, Germany) and GAPDH or actin were used as loading controls. Signals were detected with a CCD-camera imaging system and quantitatively analyzed with AIDA image analyzer 4.26 software (Raytest, Germany).

### Toxicity Assays

To study the impact of SH-SY5Y cells on PBEC or hCMEC/D3 cells and vice versa, cells in co-culture were investigated for cell viability and activation of initiator caspases 3 and 7 using CellTiterGlo Assay and CaspaseGlo Assay (Promega, Germany) respectively, according to the manufacturer’s instructions. Cell viability and cytotoxicity of PBECs after treatment with various concentrations of acitretin were assessed with CellTiter 96 Aqueous non-radioactive assay (MTS reduction assay) and CytoTox 96 non-radioactive cytotoxicity assay (lactate conversion; Promega, Germany) as recommended by the manufacturer. Untreated cells were set to 100% cell viability and cells treated with DMSO were used as control to exclude effects by the solvent. LDH-release cytotoxicity assay was performed for assessing membrane integrity and values obtained for lysed cells were set to 100%.

### TEER and Permeability Analyses

Both methodologies have been described previously [36], [37]. Briefly, starting at day 6 after isolation of PBEC or at day 3 after seeding of hCMEC/D3, the transendothelial electrical resistance (TEER) was measured with an EVOM voltohmmeter (WorldPrecision Instruments, Germany) equipped with a STX-2 chopstick electrode. Barrier resistance readings were obtained for each well individually calculated by subtracting the resistance of the blank filter membrane coated with fibronectin and multiplied by the membrane area (0.33 cm^2^) to give ohm×cm^2^.

To measure the permeability of the brain endothelial cell layer, 50 µl of the supernatant of the upper compartment was replaced by sodium fluorescein solution (Sigma Aldrich, USA) diluted in ECBM, 15% FCS, 2.5 ng/mL basal fibroblast growth factor and 10 µg/mL sodium heparin (both Sigma-Aldrich, USA), penicillin/streptomycin. Mono-cultures of PBEC were compared to the co-culture and empty transwell filters coated with fibronectin were used to determine free diffusion of sodium fluorescein through the filter membrane. Permeability coefficients (P_app_) were calculated using the equation: P_app_ = (1/(A×c_0_))×(dQ/dt), where A is the surface area of the filter (0.33 cm^2^), c_0_ the initial concentration of sodium fluorescein in the donor fluid (10 µg/mL), dQ/dt the amount of sodium fluorescein passing across the cell layer in a defined time period (3 and 24 hrs). Since the treatment with acitretin was performed for 48 hours, P_app_ for sodium fluorescein was calculated for the same time period to exclude a potential paracellular transport during longer treatment periods. 50 µl samples from the lower compartment were diluted with 1 mM NaOH and the fluorescence was measured using a multiplate reader (GeniusPuls, Tecan, Switzerland) with an excitation wavelength of λ = 480 nm and an emission wavelength of λ = 535 nm.

The permeability coefficient of acitretin was calculated as described for sodium fluorescein using the concentration of acitretin in the lower compartment determined by HPLC.

### Statistical Analyses

T-test and One-way ANOVA with Bonferroni post-test analyses were performed using GraphPad Prism version 5.00 software (Prism, USA).

## Results

### Characterization of PBECs in the BBB Co-culture Model

**Figure 1:**
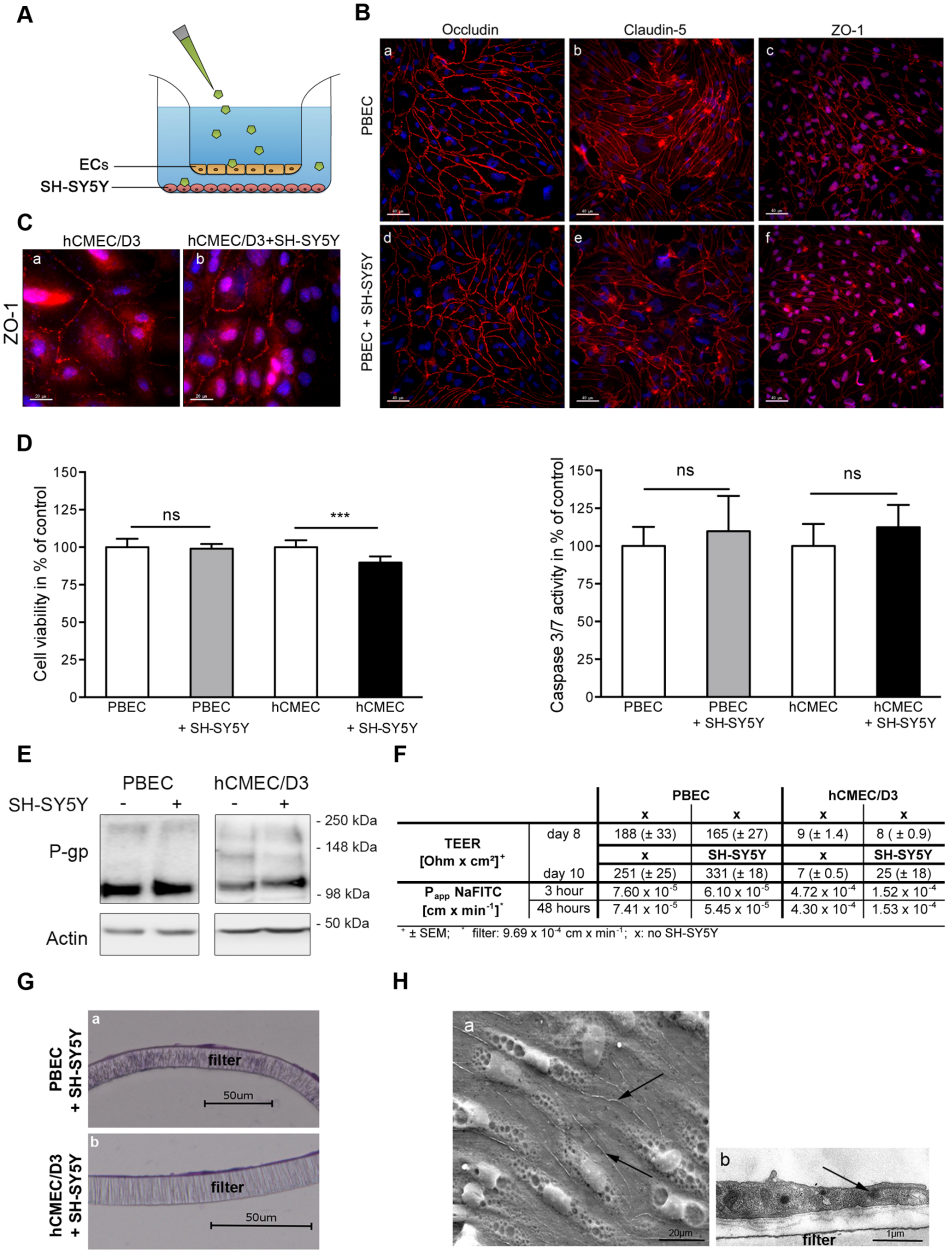
Characterization of the bio-assay model consisting of brain endothelial cells and SH-SY5Y cells. (A) Scheme of the developed bio-assay model system. (B) Immunofluorescence staining of different tight junction proteins expressed in PBEC mono- and co-culture with SH-SY5Y cells. Scale bars: 40 µm. (C) Immunofluorescent staining of zonula occludens protein (ZO-1) in hCMEC/D3 in mono-culture and in co-cultivation with SH-SY5Y cells. Scale bars: 20 µm. (D) Cell viability and caspase 3/7 activity of brain endothelial cells cultured with or without SH-SY5Y cells. Values obtained for mono-cultures were set to 100%, data represent mean ± standard deviation of three experiments (n ≥8; One Way Anova; Bonferroni post-test; ns: p>0.05; ***: p<0.001). (E) Western Blot analysis of P-gp expressed in brain endothelial cells in mono- and co-culture with SH-SY5Y. (F) Comparison of TEER and permeability coefficients (P_app_) of hCMEC/D3 and PBECs grown with or without SH-SY5Y cells. At day eight SH-SY5Y cells were seeded on the bottom of the 24-well plate. P_app_ of sodium fluorescein was determined 3 hours and 48 hours after seeding of SH-SY5Y cells. (G) HE-staining of brain endothelial cell monolayers grown on top of the filter membranes. Scale bar: 50 µm. (H) Electron microscopy images (a: SEM; b: TEM) of PBEC grown on top of the filter membranes. Arrows indicate the cell-cell connections. Scale bars: 20 µm (a) and 1 µm (b).

The development of the co-culture model system of barrier-building brain endothelial cells with neuronal cells serving as a biological reporter system is schematically shown in Figure 1 A. We initially compared two endothelial cell types for this model: immortalized hCMEC/D3 and primary porcine brain endothelial cells (PBEC). Properties of the barrier such as electrical resistance, expression of tight junction proteins and viability were assessed in mono- as well as in co-culture with SH-SY5Y cells.

After the isolation and seeding of PBECs onto filter membranes, cells formed a dense cell layer and tight junction proteins such as occludin and claudin-5 were highly expressed, as demonstrated by fluorescent staining (Figure 1 B (a–c)). In co-culture with the human neuroblastoma cell line SH-SY5Y the morphology of the PBECs was unaltered and no negative impact regarding the expression of the tight junction proteins was observed (Figure 1 B (d–f)). Cell viability and apoptosis assays confirmed that co-cultivation of PBECs with SH-SY5Y cells did not induce cytotoxicity or the activation of initiator caspases, which might negatively influence the properties of the barrier (Figure 1 D). In contrast, co-cultivation of hCMEC/D3 with SH-SY5Y cells resulted in a significant decrease in viability of endothelial cells to 89% as compared to hCMEC/D3 in mono-culture, although caspase 3 and 7 were not activated (Figure 1 D).

Western Blot analysis indicated an unaltered expression of P-glycoprotein (P-gp), the most important efflux transporter of the BBB [38], [39], in both co-culture models compared to the respective monocultures (Figure 1 E). To further characterize the endothelial cells in the co-culture model, scanning and transmission electron microscopic analyses (SEM, TEM) were performed (Figure 1 H (a–b)). Microscopic images displayed a dense monolayer and tight cell-cell contacts of PBECs grown on a filter membrane under co-culture conditions (Figure 1 H, cell-cell contacts are indicated by arrows). These results are in accordance with the strong expression of tight junction proteins as described above (Figure 1 B (a–f)). Hematoxylin-eosin (HE) staining of PBECs cross sections showed that even for a larger section of the filter membrane cells grew in a flat monolayer and continuously covered the filter membrane (Figure 1 G (a)). Although hCMEC/D3 also displayed a flat monolayer (Figure 1 G (b)), the expression of tight junction proteins was less intense and more discontinuous as compared to the primary PBECs (Figure 1 C).

The results obtained for tight junction structure are in accordance with measurement of the electrical resistance and determination of the permeability coefficients (Figure 1 F): a 20-fold higher TEER was observed for PBECs as compared to hCMEC/D3 already at the beginning of the co-cultivation with SH-SY5Y cells (day 8 and 5, respectively). hCMEC/D3 cells in general built a less dense cell layer, which was also demonstrated by the determination of the P_app_-value for sodium-fluorescein (7.41×10^5^ versus 4.3×10^4^ cm/min; Figure 1 F). Co-culture of PBECs with SH-SY5Y cells further improved the barrier properties: the mean TEER of PBECs co-cultured for 48 hrs with SH-SY5Y was 331±18 Ohms×cm^2^ and thus 32% higher than the resistance measured for PBECs in mono-culture at day 10 (Figure 1 F). This is in accordance with the observation that PBECs in co-culture with SH-SY5Y cells also possessed the lowest permeability coefficient after 48 hours of incubation in contrast to all other conditions (Figure 1 F).

In summary, these results demonstrate a continuous tightness and a strong integrity of the barrier generated by PBECs under co-cultivation conditions with SH-SY5Y cells. Therefore, PBECs were used for further analysis of the co-culture model.

### Impact of Brain Endothelial Cells on SH-SY5Y Cell Viability and APP Metabolism

**Figure 2:**
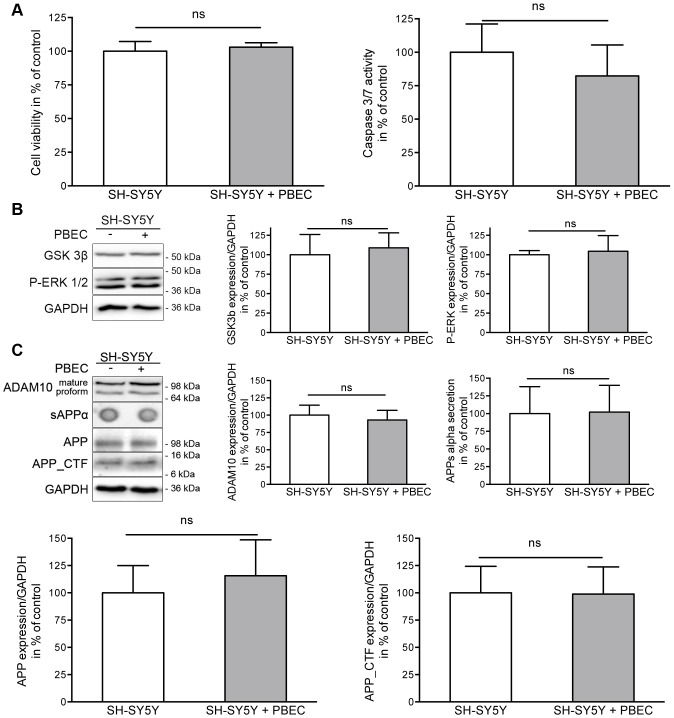
Impact of brain endothelial cells on SH-SY5Y cell viability, signal transduction and APP metabolism. (A) Cell viability and caspase 3/7 activity of SH-SY5Y cultured with or without porcine brain endothelial cells. Respective values for SH-SY5Y mono-cultures were set to 100%. (B) GSK3β and P-ERK-1/2 expression level in SH-SY5Y cells. Cells were grown in mono-cultures or co-cultivated with PBECs for 48 hrs. Protein levels were determined by Western blot and obtained values were normalized to expression of GAPDH. Values measured within mono-cultures were set to 100%. (C) ADAM10-dependent APP metabolism in SH-SY5Y cells under co-cultivation. Expression level of ADAM10, APP and APP C-terminal fragments were determined by Western blot. Values were normalized to GAPDH and set in relation to respective SH-SY5Y mono-cultures. Amount of secreted APPs-alpha was examined by a dot blot method with the specific APP N-terminal antibody (6E10). Values obtained for mono-cultured SH-SY5Y cells were set to 100% for all analysis. (three experiments; n≥6; unpaired two-tailed t-test; ns: p>0.05).

To analyze the influence of PBECs on SH-SY5Y cells under co-cultivation conditions, cell viability and potential apoptotic effects were determined. Furthermore, since the newly developed model was established for the evaluation of AD therapeutics, we had to ensure that pivotal cellular pathways, such as APP processing and AD-relevant signal transduction, were not affected by co-cultivation conditions. PBECs demonstrated no significant impact on the viability of SH-SY5Y cells after co-cultivation for 48 hours (Figure 2 A). Moreover, no induction of the initiator caspases 3 and 7 was observed in the neuronal cell line (Figure 2 A). Since glycogen synthase kinase 3-beta (GSK3- beta) is known to play a crucial role in the pathology of AD by hyperphosphorylation of tau protein [40] and by influencing the BACE1-mediated cleavage of APP [41], it is of great importance that the activity of GSK3-beta in SH-SY5Y is not altered during co-cultivation with PBECs as shown in Figure 2 B (109% compared to SH-SY5Y mono-cultures). ERK-1/−2 provide another central signaling pathway contributing to the regulation of AD-relevant proteins such as ADAM10 [42]. In our co-culture model no impact of PBECs on ERK-phosphorylation was observed (105% compared to SH-SY5Y mono-cultures, Figure 2 B). Therefore, it can be concluded that kinase activity is not altered under these conditions. Next, we showed that ADAM10 expression as well as APP expression and metabolism was not disturbed in the presence of the brain endothelial cells: SH-SY5Y cells maintained with PBECs for 48 hours neither displayed a deviating amount or maturation of ADAM10 (Figure 2 C) nor an altered APP expression. In addition, APPs-alpha secretion as well as the amount of the C-terminal fragments (APP-CTFs) were comparable to the amount found in SH-SY5Y cells kept under mono-culture conditions (Figure 2 C).

### The Effect of Acitretin on the BBB Built by PBECs

**Figure 3:**
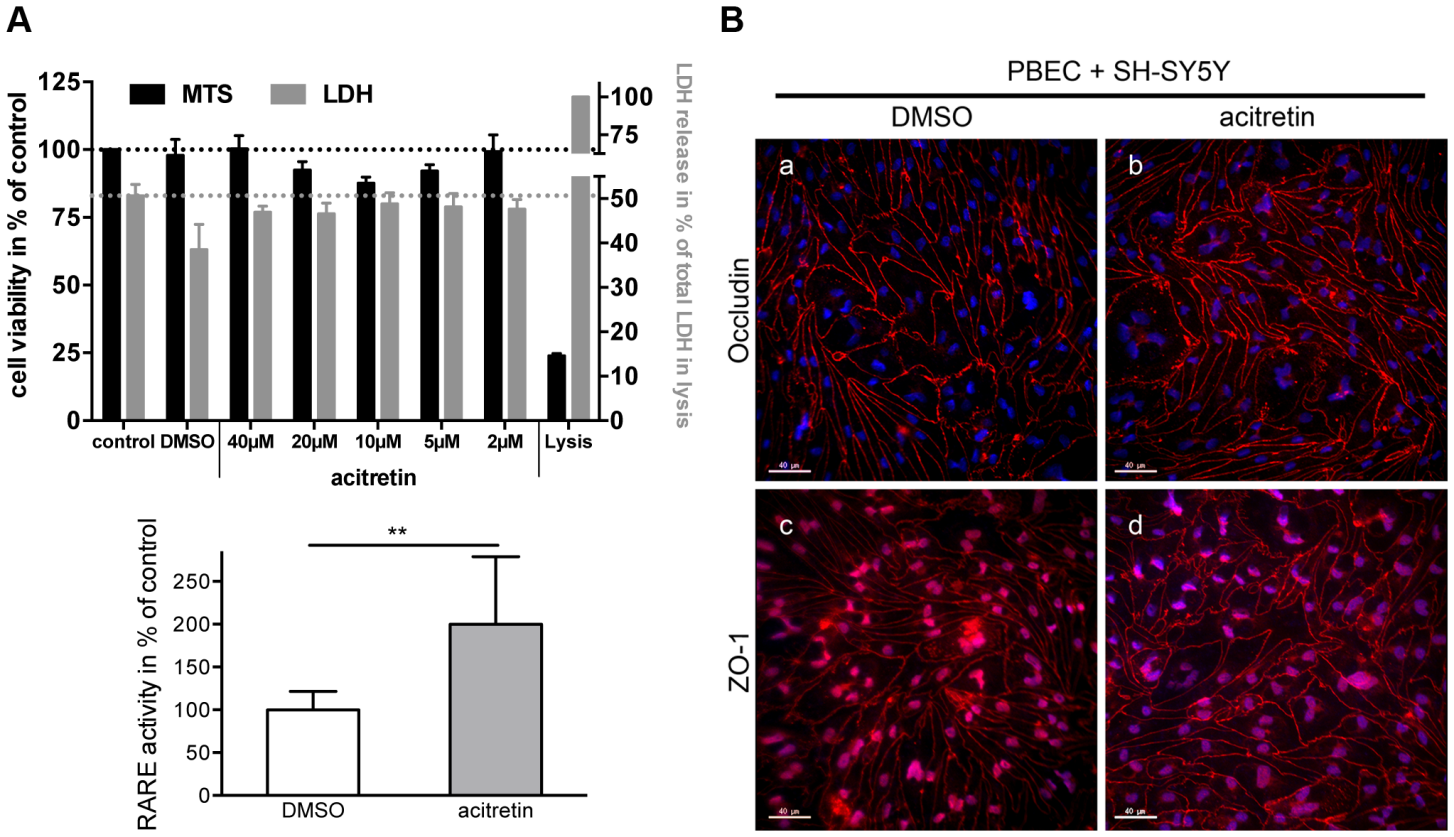
Influence of acitretin on PBEC viability and barrier properties. (A) Cell viability and cytotoxicity was measured after treatment with different concentrations of acitretin. Maximum amount of LDH (lysis) was set to 100% for the cytotoxicity assay whereas the cell viability of untreated cells (control) was set to 100%. DMSO was used as solvent control. (B) Impact of acitretin on the expression of tight junction proteins in PBEC grown on filter membranes and co-cultivated with SH-SY5Y cells. Scale bar: 40 µm. (C) Internalization of acitretin into PBEC. PBEC transfected with a luciferase-based retinoic acid response element (RARE)-containing reporter plasmid were treated with 2 µM acitretin for 48 hours and the retinoic acid dependent expression of luciferase was determined by luminescence measurement (three experiments; n = 10; unpaired two-tailed t-test; **: p<0.005).

The aim of the developed *in vitro* BBB model was to determine if a substance with already known therapeutic potential targeting AD can overcome the barrier built by brain endothelial cells. As a model drug we used acitretin, which is known to cross the BBB *in vivo* [13], [14] and has therapeutic activity by increasing the expression of the alpha-secretase ADAM10 in AD model mice [12]. To avoid misinterpretation of the *in vitro* transport data the tightness of the barrier has to be guaranteed during the treatment with acitretin. Therefore, we investigated cell viability and cytotoxicity in PBEC after treatment with different acitretin concentrations for 48 hrs. The results obtained by cytotoxicity- and LDH-assay show that acitretin did not significantly affect PBECs in any of the tested concentrations (Figure 3 A). In addition, the expression of representative tight junction proteins such as occludin and ZO-1 revealed that acitretin did not lead to a disruption of cell-cell contacts and thus the endothelial cell barrier in the co-culture model remained intact (Figure 3 B). The distinct transport mechanisms of acitretin across the BBB are not yet known. An uptake of acitretin into endothelial cells would support the assumed transcellular transport mechanism across the barrier. Thus, we transfected PBECs with a reporter plasmid containing a retinoid response element (RARE). Acitretin displaces all-trans retinoic acid from its cellular binding protein (CRABP) due to its higher affinity and therefore enhances effects based on retinoic acid receptors [43]. In response to acitretin treatment the retinoic acid-dependent expression of the reporter luciferase was increased 2-fold in PBECs compared to control cells treated with the solvent (Figure 3 C). Thus, the uptake of acitretin into PBECs was demonstrated and a potential transport across the cells can be assumed.

### Transport of Acitretin Across the Endothelial Barrier and Induction of ADAM10-Promoter Activity in Neuronal Cells

**Figure 4:**
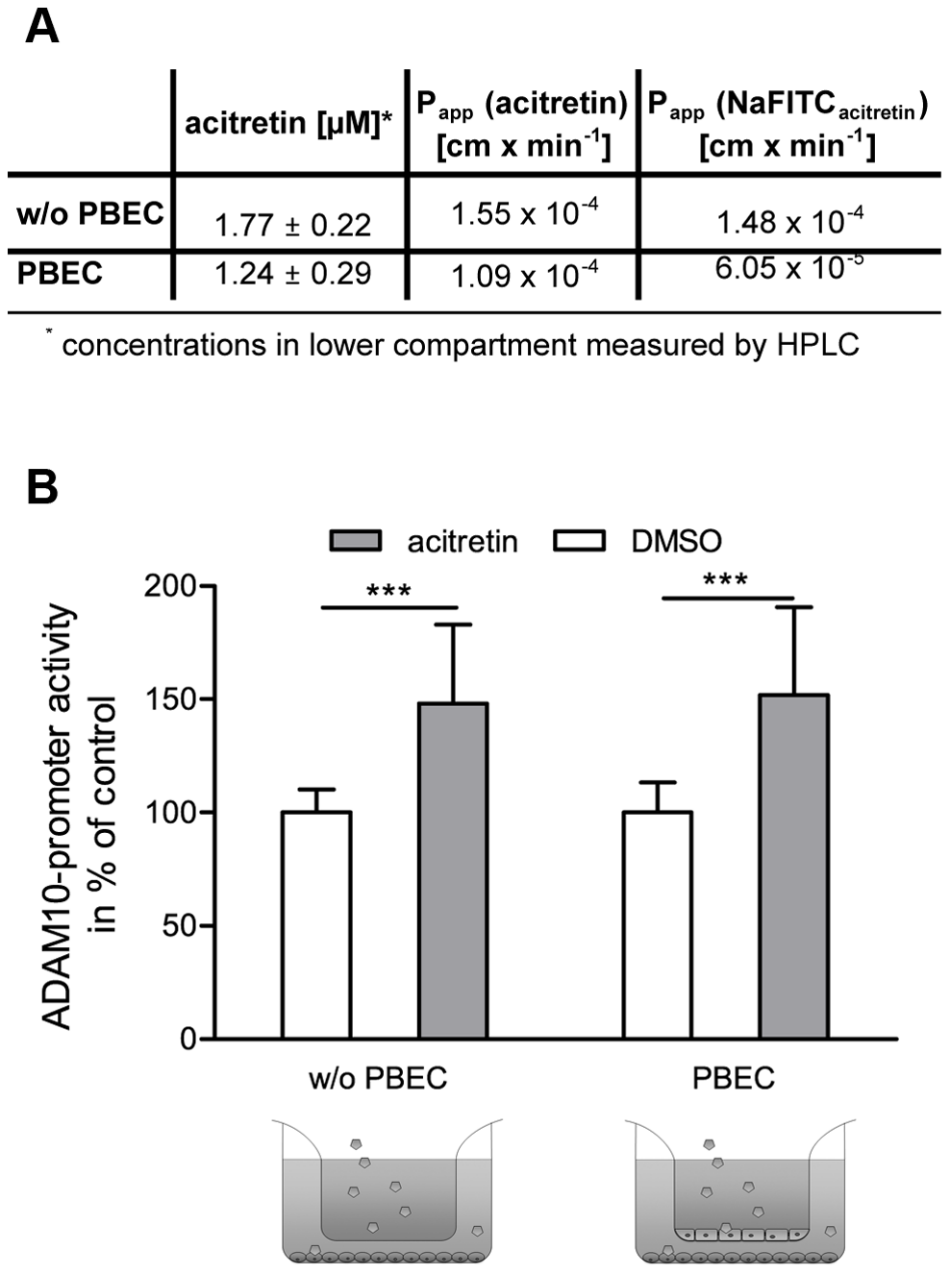
Detection of acitretin transport across the BBB. (A) After 48 hours the amount of acitretin transported across the brain endothelial cell barrier was measured by HPLC. Various concentrations were used to calculate the permeability coefficient of acitretin (P_app_ (acitretin)). To ensure the tightness of the barrier during the treatment with acitretin, the permeability coefficient of sodium fluorescein was simultaneously determined (acitretin (P_app_ (NaFITC_acitretin_)). (B) Induction of ADAM10-promoter activity in SH-SY5Y cells by acitretin transported across endothelial cells. SH-SY5Y cells were transiently transfected with an ADAM10 promoter reporter plasmid and co-cultured with PBECs for 48 hrs. Acitretin was applied to the upper compartment of the transwell system. The induction of ADAM10 promoter activity by acitretin was monitored by measurement of luciferase activity and was normalized to protein content of whole cell lysate. As control, filters without PBEC (w/o PBEC) were used (three experiments; n ≥10; One Way Anova; Bonferroni post-test; ***: p<0.001).

It was demonstrated in the experiments described above that acitretin is internalized into the porcine brain endothelial cells and does not negatively influence properties of the cellular barrier. Next, the amount of acitretin transported across the barrier was determined by HPLC after 48 hours of incubation. In parallel, we analyzed the transport properties of sodium fluorescein during acitretin treatment and calculated both, the permeability coefficient of acitretin and sodium fluorescein. The permeability coefficient of sodium fluorescein was not affected during acitretin treatment (Figure 4 A) compared to the untreated control (Figure 1 F). Furthermore, less sodium fluorescein was transported with PBECs as compared to empty filter membranes. The data suggest that under these conditions a superficial paracellular transport can be excluded. Acitretin on the contrary was transported across the brain endothelial cell barrier. The permeability coefficient of acitretin demonstrates that the transport of acitretin across the barrier was comparable to the transport across the filter membrane without PBECs (Figure 4 A). In addition, we determined the amount of transported acitretin into the lower compartment of the transwell system by HPLC. The measured acitretin concentration of 1.77±0.22 µM without endothelial cells on the filter membrane is in accordance with the theoretically obtainable concentration of 2 µM acitretin by assuming an entire substance transport. In comparison, significantly less transported acitretin was obtained in the co-culture model with endothelial cells (1.24±0.29 µM, p = 0.0269, Figure 4 A). Under these conditions, incubation with acitretin led to induction of ADAM10-promoter driven luciferase expression in SH-SY5Y cells seeded in the lower compartment of the transwell system. Elevation of promoter activity was indistinguishable from that of neuroblastoma cells cultivated without PBECs (148% versus 151%, Figure 4 B) and comparable to a promoter induction of about 150% reported previously [12].

## Discussion

Alzheimer’s disease (AD) is characterized histopathologically by neurofibrillary tangles, which occur intracellularly in neurons of AD-patients and by senile plaque deposits consisting mainly of A-beta peptides. The latter are generated by amyloidogenic processing of amyloid precursor protein (APP) by beta-secretase activity [44], [45], [46]. Alternatively APP can be cleaved by the alpha-secretase ADAM10 within the A-beta stretch, consequently preventing the release of toxic A-beta peptides [11], [47]. In addition, APP processing by ADAM10 generates a neuroprotective, soluble APP-derived fragment - sAPP-alpha - which is correlated to the survival of neurons [10], [48]. Thus, the induction of ADAM10 gene expression provides a promising approach in AD-therapy. The synthetic retinoid, acitretin, is capable of inducing the potentially AD-attenuating enzyme ADAM10 in neuronal cells and AD model mice [12]. This FDA approved drug has been previously used for systematic application in skin disease. Testing such an established drug for the ability to overcome the physiological barrier of the BBB and to potentially reach a CNS target may be a short cut to developing a novel therapy for a brain disease.

### Characterization of Brain Endothelial Cells in the BBB Co-culture Model

To make our bio-assay system, consisting of brain endothelial cells and the reporter-transfected SH-SY5Y cells, easily available to other laboratories, we first investigated if the cell line hCMEC/D3 is suitable instead of more physiological primary cells. As demonstrated by different groups the well-characterized hCMEC/D3 display brain endothelial cell characteristics, i.e. expression of brain endothelial cell specific transporters, receptors and tight junction proteins [33], [49], [50], [51]. The development of a BBB *in vitro* model has been described based on these cells [33], [52] and it has been suggested that the co-culture with a second cerebral cell type is of advantage [51]. Hatherell and colleagues reported an improvement of the barrier properties as measured by transendothelial electrical resistance (TEER) when hCMEC/D3 were co-cultivated with the human cerebral astrocyte cell line SC1810 [30]. In short duration experiments these results were in accordance with our observations, although a neuronal cell line was used instead of astrocytes. However, we aimed at developing an *in vitro* model applicable for long-term treatment. In our investigations only primary endothelial cells were able to maintain a constant and consistent tightness of the barrier during 48 hrs treatment period. Another option to improve the barrier properties of hCMEC/D3 is the use of glucocorticoids, i.e. hydrocortisone [31]. Our model system was set up to study the induction of ADAM10-promoter activity by acitretin as a model substance. Although it has been demonstrated in Tg2576 mice that ADAM10 level did not change after corticosteroid treatment [53], induction of corticoid receptors might carry the risk of potential side effects regarding other neuronal targets. For example, it has been shown in the same study that the amount of soluble A-beta 40 in the brain increased following administration of glucocorticoids. Moreover, Cury et al. revealed that glucocorticoids induce the expression of several matrix metalloproteinases and alter the activity of glycogen synthase kinase 3-beta (GSK3-beta) protein [54]. Due to the variety of potential interaction sites of hydrocortisone on the amyloid precursor protein (APP) metabolism, hydrocortisone was not applied in our model and as a consequence primary endothelial cells were used for the further experiments, based on the pronounced barrier properties exhibited by these cells.

Several co-culture models of the BBB exist, in which brain endothelial cells were co-cultured with other cell types. An overview of the variety of *in vitro* models is given by Deli et al. [55]. For example, *in vitro* co-culture models have been described consisting of brain endothelial cells and cells that are in close contact *in situ*, e.g. astrocytes or pericytes [30], [56]. It has been shown that BBB models using astrocytes improved the barrier properties regarding tightness and expression of TJ proteins [57], [58]. To our knowledge, there are only few publications examining the interaction of endothelial cells and neuronal cells, but the improvement of the barrier properties observed in our study is in accordance with those demonstrated under similar co-culture conditions [59], [60], [61].

We found TEER values of approximately 190 Ohm×cm^2^ which were higher than data published by others [55], [62], [63] and even reached values as high as 331 Ohm×cm^2^ in co-culture with SH-SY5Y cells. On the basis of *in vivo* measurements it is estimated that TEER of brain parenchymal microvessels exceeds 1000 Ohm×cm^2^ [55], [64]. In addition, determination of the permeability coefficient of sodium fluorescein revealed that the barrier built by PBECs was sufficient to prevent a paracellular transport of even small molecules. In further experiments we were able to show that the expression of tight junction proteins was not affected during co-cultivation conditions. Since at the BBB several active transport mechanisms for drug delivery are involved, we analyzed the expression of the most prominent representative of ABC transporters –P-glycoprotein (P-gp) [38], [39]. Analysis revealed that expression level of P-gp was not affected during co-culture conditions as compared to mono-cultures of endothelial cells. In addition, transmission electron microscopy (TEM) and hematoxylin-eosin (HE) staining were used to demonstrate that PBECs grew as monolayers during transport experiments.

### Impact of Brain Endothelial Cells on SH-SY5Y Cell Viability and APP Metabolism

Together with the characterization of the barrier formed by primary endothelial cells we evaluated if a co-cultivation with neuronal SH-SY5Y cells would influence barrier properties. It was demonstrated that co-cultivation with PBECs does not affect cell viability and also the activity of initiator caspases 3 and 7 in neuronal cells.

It has been postulated that extracellular receptor kinase (ERK) dysregulation plays a critical role in the development of AD. ERK is activated by oxidative stress [65], which is directly correlated to neuronal loss during disease progression. It has been demonstrated that a hyperactivation can be observed in neurons that display oxidative damage and contain hyperphophorylated tau protein [66]. Furthermore, treatment of primary cortical neurons with A-beta peptides in combination with inducers of oxidative stress, such as Fe (II), led to a rapid activation of ERK, implicating a strong correlation with AD pathogenesis [67]. In the present studies, quantitation of ERK phosphorylation by Western blot revealed no significant change during co-culture as compared to the respective mono-culture. Another regulatory protein contributing to AD pathology is the glycogen synthase kinase 3-beta (GSK3-beta), which phosphorylates tau [40], [68]. It is confirmed by several reports, that the occurrence of the tau protein in its hyperphosphorylated state is paralleled by e.g. a reduction of dendritic spines and alteration of spine morphology [69]. Western blot analysis indicated that the protein level of active GSK3-beta was not affected in co-cultures of PBECs and SH-SY5Y cells as compared to respective mono-cultures of neuronal cells.

Since the developed co-culture model aims at analyzing drugs with therapeutic potential with respect to an enhancement of ADAM10 expression, it is important to assess the endogenous ADAM10-dependent APP metabolism in co-cultivated neuronal cells. Therefore, we quantified protein levels of APP and the respective cleavage products (APP C-terminal fragments and the ADAM10-dependent soluble fragment sAPP-alpha). The amount of the secretase ADAM10– both the pro-form and the mature protein - was not affected under co-cultivation conditions. In addition, the substrate APP was not affected regarding expression and/or cleavage as demonstrated by unchanged sAPP-alpha production and CTFs.

### The Effect of Acitretin as Model Substance on the BBB Formed by PBECs

Acitretin, an aromatic retinoid able to overcome the BBB and to induce ADAM10 expression, was used to evaluate the co-culture model. Franke et al. studied the transport of retinoids across an *in vitro* BBB model using PBECs and speculated that retinoids were internalized into the ECs and released to the lower compartment [70]. To exclude a paracellular transport of acitretin in the present model we studied the transport of sodium fluorescein in parallel to the retinoid. Both substances have a similar molecular weight (acitretin: 326 Da and NaFITC: 332 Da) but fluorescein can cross the endothelial cell layer only paracellularly. We obtained a low permeability coefficient for fluorescein but high permeability coefficients for acitretin. This might indicate a transcellular transport of acitretin across the barrier. In addition, the results highlight that acitretin does not negatively influence barrier properties, as demonstrated by detection of representative tight junction protein expression using an immunofluorescence staining method as compared to solvent-treated cells. To further substantiate our results regarding transcellular transport of acitretin, we demonstrated the acitretin-induced response of PBECs transfected with a retinoid-responsive element containing a luciferase reporter vector. Acitretin liberates endogenous retinoic acid from its respective binding protein, which can only be mediated by cellular uptake of acitretin.

### Transport of Acitretin Across the Endothelial Barrier and Induction of ADAM10-Promoter Activity in Neuronal Cells

In the presented model acitretin induced ADAM10-promoter activity to 150% compared to mock-treated cells. This was previously described in a similar way without using a BBB-based model [12]. With the described model, drugs which have been demonstrated to enhance the amount of ADAM10 and consequently display therapeutic potential regarding AD can subsequently be examined for their ability to cross the BBB by a sensitive promoter-dependent luciferase assay. This avoids the need for different HPLC applications and also reduces the number of animals for *in vivo* evaluation of BBB permeability. Furthermore, this bio-assay can easily be modified to analyze the influence of unique compounds on other disease relevant proteins by adapting the respective promoter reporter assay. This might consequently be useful to evaluate BBB penetrance of drugs against brain diseases or for investigating modification of drugs to obtain or enhance BBB permeability, such as coating on or attaching to nanoparticles. Moreover, our model might also be useful for unraveling transport mechanism for e.g. acitretin or for analysis of drug-drug interference with already applied AD symptomatic treatments such as Donepezil.

## Conclusion

The results presented in this study demonstrate the functionality of a novel bio-assay system. The well-characterized *in vitro* model is suitable for predicting drug passage across the BBB of therapeutically useful drugs applicable for AD. This model uses an easy and fast reporter assay read-out system present in neuronal co-cultured cells which might also be adapted for drugs with different target genes.

## References

[1] Hendrie HC (1998) Epidemiology of dementia and Alzheimer’s disease. Am J Geriatr Psychiatry 6: S3–18. 9581216 10.1097/00019442-199821001-00002

[2] Bowman GL, Quinn JF (2008) Alzheimer’s disease and the Blood-Brain Barrier: Past, Present and Future. Aging health 4: 47–55. 19924258 10.2217/1745509X.4.1.47 PMC2778025

[3] Maruszak A, Zekanowski C (2011) Mitochondrial dysfunction and Alzheimer’s disease. Prog Neuropsychopharmacol Biol Psychiatry 35: 320–330. 20624441 10.1016/j.pnpbp.2010.07.004

[4] Iqbal K, Grundke-Iqbal I (2008) Alzheimer neurofibrillary degeneration: significance, etiopathogenesis, therapeutics and prevention. J Cell Mol Med 12: 38–55. 18194444 10.1111/j.1582-4934.2008.00225.x PMC3139457

[5] Endres K, Fahrenholz F (2010) Upregulation of the alpha-secretase ADAM10–risk or reason for hope? FEBS J 277: 1585–1596. 20136654 10.1111/j.1742-4658.2010.07566.x

[6] Sathya M, Premkumar P, Karthick C, Moorthi P, Jayachandran KS, et al (2012) BACE1 in Alzheimer’s disease. Clin Chim Acta 414: 171–178. 22926063 10.1016/j.cca.2012.08.013

[7] Wolfe MS (2012) gamma-Secretase as a target for Alzheimer’s disease. Adv Pharmacol 64: 127–153. 22840746 10.1016/B978-0-12-394816-8.00004-0

[8] Mattson MP (1997) Cellular actions of beta-amyloid precursor protein and its soluble and fibrillogenic derivatives. Physiol Rev 77: 1081–1132. 9354812 10.1152/physrev.1997.77.4.1081

[9] Caille I, Allinquant B, Dupont E, Bouillot C, Langer A, et al (2004) Soluble form of amyloid precursor protein regulates proliferation of progenitors in the adult subventricular zone. Development 131: 2173–2181. 15073156 10.1242/dev.01103

[10] Thornton E, Vink R, Blumbergs PC, Van Den Heuvel C (2006) Soluble amyloid precursor protein alpha reduces neuronal injury and improves functional outcome following diffuse traumatic brain injury in rats. Brain Res 1094: 38–46. 16697978 10.1016/j.brainres.2006.03.107

[11] Postina R, Schroeder A, Dewachter I, Bohl J, Schmitt U, et al (2004) A disintegrin-metalloproteinase prevents amyloid plaque formation and hippocampal defects in an Alzheimer disease mouse model. The Journal of clinical investigation 113: 1456–1464. 15146243 10.1172/JCI20864 PMC406531

[12] Tippmann F, Hundt J, Schneider A, Endres K, Fahrenholz F (2009) Up-regulation of the alpha-secretase ADAM10 by retinoic acid receptors and acitretin. FASEB J 23: 1643–1654. 19144697 10.1096/fj.08-121392

[13] Eisenhardt EU, Bickel MH (1994) Kinetics of tissue distribution and elimination of retinoid drugs in the rat. I. Acitretin. Drug Metab Dispos 22: 26–30. 8149885

[14] Holthoewer D, Endres K, Schuck F, Hiemke C, Schmitt U, et al (2012) Acitretin, an enhancer of alpha-secretase expression, crosses the blood-brain barrier and is not eliminated by P-glycoprotein. Neurodegener Dis 10: 224–228. 22301853 10.1159/000334300

[15] Kelleher RJ, Soiza RL (2013) Evidence of endothelial dysfunction in the development of Alzheimer’s disease: Is Alzheimer’s a vascular disorder? Am J Cardiovasc Dis 3: 197–226. 24224133 PMC3819581

[16] Zipser BD, Johanson CE, Gonzalez L, Berzin TM, Tavares R, et al (2007) Microvascular injury and blood-brain barrier leakage in Alzheimer’s disease. Neurobiol Aging 28: 977–986. 16782234 10.1016/j.neurobiolaging.2006.05.016

[17] Karch A, Manthey H, Ponto C, Hermann P, Heinemann U, et al (2013) Investigating the Association of ApoE Genotypes with Blood-Brain Barrier Dysfunction Measured by Cerebrospinal Fluid-Serum Albumin Ratio in a Cohort of Patients with Different Types of Dementia. PLoS ONE 8: e84405. 24386372 10.1371/journal.pone.0084405 PMC3874026

[18] Vos SJ, Xiong C, Visser PJ, Jasielec MS, Hassenstab J, et al (2013) Preclinical Alzheimer’s disease and its outcome: a longitudinal cohort study. Lancet Neurol 12: 957–965. 24012374 10.1016/S1474-4422(13)70194-7 PMC3904678

[19] Hawkins BT, Davis TP (2005) The blood-brain barrier/neurovascular unit in health and disease. Pharmacological reviews 57: 173–185. 15914466 10.1124/pr.57.2.4

[20] Löscher W, Potschka H (2005) Role of drug efflux transporters in the brain for drug disposition and treatment of brain diseases. Progress in neurobiology 76: 22–76. 16011870 10.1016/j.pneurobio.2005.04.006

[21] Abbott NJ, Patabendige AAK, Dolman DEM, Yusof SR, Begley DJ (2010) Structure and function of the blood-brain barrier. Neurobiology of Disease: 13–25. 10.1016/j.nbd.2009.07.030 19664713

[22] Dehouck MP, Méresse S, Delorme P, Fruchart JC, Cecchelli R (1990) An easier, reproducible, and mass-production method to study the blood-brain barrier in vitro. Journal of neurochemistry 54: 1798–1801. 2182777 10.1111/j.1471-4159.1990.tb01236.x

[23] Franke H, Galla H, Beuckmann CT (2000) Primary cultures of brain microvessel endothelial cells: a valid and flexible model to study drug transport through the blood-brain barrier in vitro. Brain research Brain research protocols 5: 248–256. 10906490 10.1016/s1385-299x(00)00020-9

[24] Culot M, Lundquist S, Vanuxeem D, Nion S, Landry C, et al (2008) An in vitro blood-brain barrier model for high throughput (HTS) toxicological screening. Toxicology in vitro : an international journal published in association with BIBRA 22: 799–811. 18280105 10.1016/j.tiv.2007.12.016

[25] Patabendige A, Skinner RA, Abbott NJ (2013) Establishment of a simplified in vitro porcine blood-brain barrier model with high transendothelial electrical resistance. Brain Res 1521: 1–15. 22789905 10.1016/j.brainres.2012.06.057 PMC3694297

[26] Fricker G, Nobmann S, Miller DS (2002) Permeability of porcine blood brain barrier to somatostatin analogues. British journal of pharmacology 135: 1308–1314. 11877340 10.1038/sj.bjp.0704557 PMC1573221

[27] Lai C-H, Kuo K-H (2005) The critical component to establish in vitro BBB model: Pericyte. Brain research Brain research reviews 50: 258–265. 16199092 10.1016/j.brainresrev.2005.07.004

[28] Kido Y, Tamai I, Nakanishi T, Kagami T, Hirosawa I, et al (2004) Evaluation of blood-brain barrier transporters by co-culture of brain capillary endothelial cells with astrocytes. Drug metabolism and pharmacokinetics 17: 34–41. 10.2133/dmpk.17.34 15618650

[29] Nakagawa S, Deli MA, Nakao S, Honda M, Hayashi K, et al (2007) Pericytes from brain microvessels strengthen the barrier integrity in primary cultures of rat brain endothelial cells. Cellular and molecular neurobiology 27: 687–694. 17823866 10.1007/s10571-007-9195-4 PMC11517186

[30] Hatherell K, Couraud P-O, Romero IA, Weksler B, Pilkington GJ (2011) Development of a three-dimensional, all-human in vitro model of the blood-brain barrier using mono-, co-, and tri-cultivation Transwell models. Journal of neuroscience methods 199: 223–229. 21609734 10.1016/j.jneumeth.2011.05.012

[31] Forster C, Burek M, Romero IA, Weksler B, Couraud P-O, et al (2008) Differential effects of hydrocortisone and TNF{alpha} on tight junction proteins in an in vitro model of the human blood-brain barrier. J Physiol 586: 1937–1949. 18258663 10.1113/jphysiol.2007.146852 PMC2375735

[32] Stins MF, Badger J, Sik Kim K (2001) Bacterial invasion and transcytosis in transfected human brain microvascular endothelial cells. Microb Pathog 30: 19–28. 11162182 10.1006/mpat.2000.0406

[33] Weksler BB, Subileau EA, Perrière N, Charneau P, Holloway K, et al (2005) Blood-brain barrier-specific properties of a human adult brain endothelial cell line. The FASEB journal : official publication of the Federation of American Societies for Experimental Biology 19: 1872–1874. 16141364 10.1096/fj.04-3458fje

[34] Unger RE, Oltrogge JB, Briesen H, Engelhardt B, Woelki U, et al (2002) Isolation and molecular characterization of brain microvascular endothelial cells from human brain tumors. In Vitro Cellular & Developmental Biology - Animal 38: 273–281. 12418924 10.1290/1071-2690(2002)038<0273:IAMCOB>2.0.CO;2

[35] Steiner H, Kostka M, Romig H, Basset G, Pesold B, et al (2000) Glycine 384 is required for presenilin-1 function and is conserved in bacterial polytopic aspartyl proteases. Nat Cell Biol 2: 848–851. 11056541 10.1038/35041097

[36] Hermanns MI, Kasper J, Dubruel P, Pohl C, Uboldi C, et al.. (2009) An impaired alveolar-capillary barrier in vitro: effect of proinflammatory cytokines and consequences on nanocarrier interaction. Journal of The Royal Society Interface. 10.1098/rsif.2009.0288.focus PMC2843988 19793744

[37] Kasper J, Hermanns MI, Bantz C, Maskos M, Stauber R, et al (2011) Inflammatory and cytotoxic responses of an alveolar-capillary coculture model to silica nanoparticles: comparison with conventional monocultures. Particle and fibre toxicology 8: 6. 21272353 10.1186/1743-8977-8-6 PMC3040689

[38] Cordon-Cardo C, O’Brien JP, Casals D, Rittman-Grauer L, Biedler JL, et al (1989) Multidrug-resistance gene (P-glycoprotein) is expressed by endothelial cells at blood-brain barrier sites. Proc Natl Acad Sci U S A 86: 695–698. 2563168 10.1073/pnas.86.2.695 PMC286540

[39] Sharom FJ (2011) The P-glycoprotein multidrug transporter. Essays Biochem 50: 161–178. 21967057 10.1042/bse0500161

[40] Ishiguro K, Shiratsuchi A, Sato S, Omori A, Arioka M, et al (1993) Glycogen synthase kinase 3 beta is identical to tau protein kinase I generating several epitopes of paired helical filaments. FEBS Lett 325: 167–172. 7686508 10.1016/0014-5793(93)81066-9

[41] Ly PT, Wu Y, Zou H, Wang R, Zhou W, et al (2013) Inhibition of GSK3beta-mediated BACE1 expression reduces Alzheimer-associated phenotypes. J Clin Invest 123: 224–235. 23202730 10.1172/JCI64516 PMC3533290

[42] Wan XZ, Li B, Li YC, Yang XL, Zhang W, et al (2012) Activation of NMDA receptors upregulates a disintegrin and metalloproteinase 10 via a Wnt/MAPK signaling pathway. J Neurosci 32: 3910–3916. 22423111 10.1523/JNEUROSCI.3916-11.2012 PMC6703469

[43] Armstrong JL, Redfern CP, Veal GJ (2005) 13-cis retinoic acid and isomerisation in paediatric oncology–is changing shape the key to success? Biochem Pharmacol 69: 1299–1306. 15826600 10.1016/j.bcp.2005.02.003

[44] Sinha S, Lieberburg I (1999) Cellular mechanisms of beta-amyloid production and secretion. Proc Natl Acad Sci U S A 96: 11049–11053. 10500121 10.1073/pnas.96.20.11049 PMC34239

[45] Vassar R, Bennett BD, Babu-Khan S, Kahn S, Mendiaz EA, et al (1999) Beta-secretase cleavage of Alzheimer’s amyloid precursor protein by the transmembrane aspartic protease BACE. Science 286: 735–741. 10531052 10.1126/science.286.5440.735

[46] Hussain I, Powell D, Howlett DR, Tew DG, Meek TD, et al (1999) Identification of a novel aspartic protease (Asp 2) as beta-secretase. Mol Cell Neurosci 14: 419–427. 10656250 10.1006/mcne.1999.0811

[47] Lammich S, Kojro E, Postina R, Gilbert S, Pfeiffer R, et al (1999) Constitutive and regulated alpha-secretase cleavage of Alzheimer’s amyloid precursor protein by a disintegrin metalloprotease. Proceedings of the National Academy of Sciences of the United States of America 96: 3922–3927. 10097139 10.1073/pnas.96.7.3922 PMC22396

[48] Ishida A, Furukawa K, Keller JN, Mattson MP (1997) Secreted form of beta-amyloid precursor protein shifts the frequency dependency for induction of LTD, and enhances LTP in hippocampal slices. Neuroreport 8: 2133–2137. 9243598 10.1097/00001756-199707070-00009

[49] Carl SM, Lindley DJ, Couraud PO, Weksler BB, Romero I, et al.. (2010) ABC and SLC Transporter Expression and Pot Substrate Characterization across the Human CMEC/D3 Blood-Brain Barrier Cell Line. Molecular Pharmaceutics. 10.1021/mp900178j PMC2914114 20524699

[50] Dauchy S, Miller F, Couraud P-O, Weaver RJ, Weksler B, et al (2009) Expression and transcriptional regulation of ABC transporters and cytochromes P450 in hCMEC/D3 human cerebral microvascular endothelial cells. Biochemical Pharmacology 2009: 897–909. 10.1016/j.bcp.2008.11.001 19041851

[51] Weksler B, Romero IA, Couraud PO (2013) The hCMEC/D3 cell line as a model of the human blood brain barrier. Fluids Barriers CNS 10: 16. 23531482 10.1186/2045-8118-10-16 PMC3623852

[52] Poller B, Drewe J, Krähenbühl S, Huwyler J, Gutmann H (2010) Regulation of BCRP (ABCG2) and P-glycoprotein (ABCB1) by cytokines in a model of the human blood-brain barrier. Cellular and molecular neurobiology 30: 63–70. 19629677 10.1007/s10571-009-9431-1 PMC11498628

[53] Joshi YB, Chu J, Pratico D (2012) Stress hormone leads to memory deficits and altered tau phosphorylation in a model of Alzheimer’s disease. J Alzheimers Dis 31: 167–176. 22531419 10.3233/JAD-2012-120328 PMC3882896

[54] Cury PR, Araujo VC, Canavez F, Furuse C, Araujo NS (2007) Hydrocortisone affects the expression of matrix metalloproteinases (MMP-1, -2, -3, -7, and -11) and tissue inhibitor of matrix metalloproteinases (TIMP-1) in human gingival fibroblasts. J Periodontol 78: 1309–1315. 17608586 10.1902/jop.2007.060225

[55] Deli MA, Abraham CS, Kataoka Y, Niwa M (2005) Permeability studies on in vitro blood-brain barrier models: physiology, pathology, and pharmacology. Cell Mol Neurobiol 25: 59–127. 15962509 10.1007/s10571-004-1377-8 PMC11529645

[56] Zozulya A, Weidenfeller C, Galla H-J (2008) Pericyte-endothelial cell interaction increases MMP-9 secretion at the blood-brain barrier in vitro. Brain research 1189: 1–11. 18061148 10.1016/j.brainres.2007.10.099

[57] Gaillard PJ, Voorwinden LH, Nielsen JL, Ivanov A, Atsumi R, et al (2001) Establishment and functional characterization of an in vitro model of the blood-brain barrier, comprising a co-culture of brain capillary endothelial cells and astrocytes. European journal of pharmaceutical sciences : official journal of the European Federation for Pharmaceutical Sciences 12: 215–222. 11113640 10.1016/s0928-0987(00)00123-8

[58] Zhang Y, Li CSW, Ye Y, Johnson K, Poe J, et al (2006) Porcine brain microvessel endothelial cells as an in vitro model to predict in vivo blood-brain barrier permeability. Drug metabolism and disposition: the biological fate of chemicals 34: 1935–1943. 16896068 10.1124/dmd.105.006437

[59] Schiera G, Bono E, Raffa MP, Gallo A, Pitarresi GL, et al (2003) Synergistic effects of neurons and astrocytes on the differentiation of brain capillary endothelial cells in culture. J Cell Mol Med 7: 165–170. 12927055 10.1111/j.1582-4934.2003.tb00215.x PMC6740229

[60] Tontsch U, Bauer HC (1991) Glial cells and neurons induce blood-brain barrier related enzymes in cultured cerebral endothelial cells. Brain Res 539: 247–253. 1675906 10.1016/0006-8993(91)91628-e

[61] Weidenfeller C, Svendsen CN, Shusta EV (2007) Differentiating embryonic neural progenitor cells induce blood-brain barrier properties. J Neurochem 101: 555–565. 17254017 10.1111/j.1471-4159.2006.04394.x PMC2657050

[62] Igarashi Y, Utsumi H, Chiba H, Yamada-Sasamori Y, Tobioka H, et al (1999) Glial cell line-derived neurotrophic factor induces barrier function of endothelial cells forming the blood-brain barrier. Biochem Biophys Res Commun 261: 108–112. 10405331 10.1006/bbrc.1999.0992

[63] Yamagata K, Tagami M, Takenaga F, Yamori Y, Nara Y, et al (2003) Polyunsaturated fatty acids induce tight junctions to form in brain capillary endothelial cells. Neuroscience 116: 649–656. 12573708 10.1016/s0306-4522(02)00715-7

[64] Gumbleton M, Audus KL (2001) Progress and limitations in the use of in vitro cell cultures to serve as a permeability screen for the blood-brain barrier. J Pharm Sci 90: 1681–1698. 11745727 10.1002/jps.1119

[65] McCubrey JA, Lahair MM, Franklin RA (2006) Reactive oxygen species-induced activation of the MAP kinase signaling pathways. Antioxid Redox Signal 8: 1775–1789. 16987031 10.1089/ars.2006.8.1775

[66] Perry G, Roder H, Nunomura A, Takeda A, Friedlich AL, et al (1999) Activation of neuronal extracellular receptor kinase (ERK) in Alzheimer disease links oxidative stress to abnormal phosphorylation. Neuroreport 10: 2411–2415. 10439473 10.1097/00001756-199908020-00035

[67] Kuperstein F, Yavin E (2002) ERK activation and nuclear translocation in amyloid-beta peptide- and iron-stressed neuronal cell cultures. Eur J Neurosci 16: 44–54. 12153530 10.1046/j.1460-9568.2002.02056.x

[68] Ishiguro K, Takamatsu M, Tomizawa K, Omori A, Takahashi M, et al (1992) Tau protein kinase I converts normal tau protein into A68-like component of paired helical filaments. J Biol Chem 267: 10897–10901. 1587865

[69] Messing L, Decker JM, Joseph M, Mandelkow E, Mandelkow EM (2013) Cascade of tau toxicity in inducible hippocampal brain slices and prevention by aggregation inhibitors. Neurobiol Aging 34: 1343–1354. 23158765 10.1016/j.neurobiolaging.2012.10.024 PMC4984976

[70] Franke H, Galla H-J, Beuckmann CT (1999) An improved low-permeability in vitro-model of the blood-brain barrier: transport studies on retinoids, sucrose, haloperidol, caffeine and mannitol. Brain Research 818: 65–71. 9914438 10.1016/s0006-8993(98)01282-7

